# Microbiological and chemical evaluation of dairy products commercialized in the Lebanese market

**DOI:** 10.14202/vetworld.2022.2575-2586

**Published:** 2022-11-14

**Authors:** Hiyam El Kojok, Mahmoud Khalil, Rima Hage, Rola Jammoul, Adla Jammoul, Nada El Darra

**Affiliations:** 1 Department of Biological Sciences, Beirut Arab University, Faculty of Sciences, Tarik El Jedidah - Beirut, P.O. Box: 115020 Riad EL Solh 1107 2809, Lebanon; 2 Department of Food, Lebanese Agricultural Research Institute, Fanar, Lebanon P.O. Box 2611, Beirut 1107 2809, Lebanon; 3 Phytopharmacy Laboratory, Ministry of Agriculture of Lebanon, Kfarchima, Lebanon; 4 Beirut Arab University, Faculty of Health Sciences, Tarik El Jedidah - Beirut, P.O. Box: 115020 Riad EL Solh 1107 2809, Lebanon

**Keywords:** antibiotic residues, antibiotic resistance, cheese, dairy products, high-performance liquid chromatography, microbial contamination

## Abstract

**Background and Aim:**

Cheese is considered an essential component of the Lebanese table, however, several foodborne illnesses have been reported due to cheese consumption. This study aimed to assess the microbiological quality and the occurrence of antibiotic and pesticide residues in two traditional Lebanese cheeses, Akkawi and Baladiyeh. In addition, drug resistance of isolated pathogens from the cheese samples was evaluated.

**Materials and Methods:**

Fifty Akkawi and Baladiyeh cheese samples were obtained in duplicate from 37 different commercial brands in supermarkets and shops from various regions of Lebanon. Samples of different weights were either individually vacuum packed or soaked in brine unpacked where it was placed in plastic bag after being purchased. Samples were homogenized to determine antibiotic and pesticide residues using liquid and gas chromatography coupled to mass spectrometry, and microbiological evaluation was performed according to the International Organization for Standardization reference analytical methods. The disk diffusion method was used to determine the susceptibility of these isolates to antibiotics.

**Results:**

Microbiologically, 17% of Akkawi and 14% of Baladiyeh samples were found to be non-conforming. The bacterial isolates (n = 29) were tested for their susceptibility to 11 different antibiotics commonly prescribed in the Lebanese community or used for treating infections caused by Gram-negative bacteria and listeriosis. Each isolate was found to be resistant to at least three antibiotics. Liquid and gas chromatography coupled to mass spectroscopy analysis showed the absence of pesticide residues in all samples. However, sulfamethazine antibiotic residue was found in 14% of the samples.

**Conclusion:**

The results suggest that the cheese samples tested could cause foodborne illnesses due to the detection of pathogenic bacteria and are a public health concern due to the presence of antibiotic residues and the transmission of multidrug-resistant organisms.

## Introduction

According to the data from the Lebanese Ministry of Industry, the number of dairy processing units in Lebanon increased dramatically, from 64 in 1985 to 500 units in 2014, considering registered, informal, and unreported units [1]. It should be noted that several processing units operate without a sanitary license from the Ministry of Agriculture. This makes controlling the quality of Lebanese dairy products in the Lebanese market extremely difficult. Lebanese dairy processing units are classified as modern, semi-modern, or traditional dairies, and they collect milk from local farmers, collection centers, or from their farms for large industries [2]. Cheese, labneh, and yogurt are the most commonly produced dairy products in the Lebanese market.

Dairy products, particularly cheese, are valued for their rich nutrient content and are considered an important component in the Mediterranean diet, which includes a wide variety of white cheeses (Akkawi, Baladiyeh, Halloumi, and double cream) [3, 4]. However, cheese in general is a safe and nutritionally important component of the human diet. However, foodborne illnesses associated with cheese consumption have been reported in many countries, primarily because of contamination with causative agents such as *Listeria monocytogenes*, *Staphylococcus aureus*, and *Escherichia coli* [5]. Dib *et al*. [2] discovered alarming levels of *Coliforms*, *E*. *coli*, *Listeria*, and *Salmonella* in a study on the microbiological profile of Lebanese cheese. Baladi Akkawi and double cream had the highest level of non-conformity. Furthermore, Dabboussi *et al*. [6] discovered *L. monocytogenes* and *Salmonella* contamination in Akkawi cheese, randomly selected from the region of North Lebanon.

However, microorganisms are not the only threat to the safety of milk and dairy products. Chemical hazards such as veterinary drugs (antimicrobial agents), pesticides, heavy metals, and hormones could contaminate dairy products [7]. Antimicrobial drugs, primarily antibiotics, are the most controversial residues found in dairy products due to their extensive use [8]. Since their discovery, antibiotics have been used to treat both humans and animals from infectious diseases [9] as well as a growth promoter of food-producing animals [10, 11]. Antibiotics prevalently used in dairy cattle belong to five classes: Beta-lactams (e.g., penicillin and cephalosporin), tetracyclines (e.g., oxytetracycline, tetracycline, and chlortetracycline), aminoglycosides (e.g., streptomycin [S], neomycin, and gentamicin), macrolides (e.g., erythromycin [E]), and sulfonamides (e.g., sulfamethazine [SMT]) [12, 13]. The evolution of the dairy sectors around the world, and the need to increase their productivity, has resulted in increased antibiotic use [9, 14, 15], making antibiotic residues a public health hazard [16], and increasing the risk of antibiotic resistance [8] through transferring antibiotic-resistant bacterial strains across the food chain [17]. Recent studies have shown that antibiotics persist in animal-derived foods such as milk, eggs, and meat, even after heat treatment, causing, in addition to antibiotic resistance, gastrointestinal problems in addition to the development of allergies in humans [13]. The World Health Organization and the World Organization for Animal Health promoted the “One Health” concept, emphasizing the close relationship between humans, animals, and the environment, which resulted in a special health concept [18]. The main environmental concerns about antibiotic residues are that they alter the human microbiome and promote bacterial resistance, in addition to exerting selection pressure on the environmental microbiome and creating an environmental antibiotic resistance reservoir [19]. Furthermore, the presence of antibiotic residues in milk has a significant impact on the preparation of cultured products such as cheese [20] by inhibiting the starter cultures and disrupting the fermentation process of cheese [21] and influencing the biological processes responsible for unique cheese flavor [22]. Hence, information about antibiotic residues is critical for both consumers and producers. To ensure the safety of dairy products, the European Commission has established maximum residue limits (MRLs) for certain drugs in animal-derived foods [23]. Moreover, the elimination period must be properly determined, which is the time required for most antibiotic residues to be eliminated through urine. Hence, if the required elimination period is not well respected, the risk of the presence of antibiotic residues in milk and dairy products could increase. Researchers assessing antibiotic residues in dairy goats have revealed that the 7-day minimum withdrawal period stipulated by the legislation for off-label treatment is not always sufficient to ensure the total absence of drug residues in milk [24]. In addition, pesticide residues are another source of chemical contamination in dairy products, as stable pesticides can be transferred to milk after animals consume contaminated feed or drink. Currently, pesticides are being used worldwide, primarily in developed countries, to control various agricultural problems (pests, insects, and microbes) and result in increased productivity [8].

Currently, laboratory-based techniques such as chromatographic methods are used to quantify chemical contaminants in food matrixes. However, an urgent need for quick and easy methods has resulted in the development of promising alternatives, such as immunosensors that are capable of detecting residues at very low concentrations [25].

Limited studies on traditional dairy products in Lebanon have revealed high levels of bacterial contamination with *Coliforms* and *Staphylococcus* species that are resistant to at least one antimicrobial drug [26–28]. Furthermore, several studies conducted over the last decade have revealed the disturbing situation of antimicrobial resistance (AMR) and the spread of multidrug-resistant (MDR) strains because of irresponsibility, lack of knowledge, the availability of antimicrobial drugs without a prescription, and excessive and extensive drug use [14, 29].

This study evaluated the microbiological quality and the presence of antibiotic and pesticide residues in traditional Lebanese cheeses, Akkawi and Baladiyeh, in addition to the assessment of drug resistance of isolated pathogens from the cheese samples.

## Materials and Methods

### Ethical approval

No approval of research ethics committees was required to accomplish the goals of this study because the experimental study was conducted on food samples (milk and cheese).

### Study period and location

Samples were collected from January to May 2021 from supermarkets and small shops across Lebanon. Experiments were conducted at the laboratories of Beirut Arab University and Lebanese Agricultural Research Institute during the period extended from January to August 2021.

### Sampling

Twenty-five samples each of Akkawi and Baladiyeh cheese were procured in duplicate from supermarkets and small shops located in Beirut, Mount Lebanon, South Lebanon, and Bekaa. As shown in Table-1, samples were sold either individually vacuum packed (58%) or soaked in brine unpacked (42%), where it was then placed in plastic bag after being purchased. A total of 50 samples, representing 37 different brands were collected. Samples were transferred to the laboratory in a cold chain and analyzed microbiologically on the same day. Another sample from the same lot was stored at −20°C for antibiotic and pesticide residue analysis by grinding the sample followed by the extraction procedure.

**Table-1:** Sampling of dairy products (Akkawi cheese and Baladiyeh cheese).

(a) Industries’ locations among Lebanese governorate					

Cheese type	Beirut, n (%)	South Lebanon, n (%)	Bekaa, n (%)	Mount Lebanon, n (%)	Total number of samples by type
Akkawi cheese	1 (4)	3 (12)	18 (72)	3 (12)	25
Baladiyeh cheese	3 (12)	6 (24)	12 (48)	4 (16)	25
Total number of samples	4 (8)	9 (18)	30 (60)	7 (14)	50

**(b) Packaging of commercialized samples**					

**Cheese type**	**Individually vacuum packed, n (%)**		**Soaked in brine, n (%)**	**Total number of samples by type**	

Akkawi cheese	18 (72)		7 (28)	25	
Baladiyeh cheese	11 (44)		14 (56)	25	
Total number of samples	29 (58)		21 (42)	50	

### Microbiological analysis

The International Organization for Standardization (ISO) standards were used to investigate the microbiological criteria of cheese samples. Total *Coliforms* [30], *E. coli* [31], *S. aureus* [32], *L. monocytogenes* [33], and *Salmonella* [34] were identified in the sample. Table-2 provides the microbiological parameters investigated and the relative identification techniques used in accordance with ISO reference analytical method. Homogenized samples were serially diluted and inoculated on the media recommended for each tested bacterium. All media were supplied by Scharlu, Spain.

**Table-2:** Microbiological parameters investigated and relative identification technique.

Microbiological parameters	ISO reference analytical method	Medium	Incubation condition
Total *Coliforms*	ISO 4832:2006	Violet red bile agar	37°C – 24 h
*Escherichia coli*	ISO 16649–2:2001	Microinstant tryptone bile X-glucuronide agar	44°C – 24 h
*Staphylococcus aureus*	ISO 6881–1:1999	Baird parker selective agar	37°C – 24 h
*Salmonella*	ISO 6579:2002	Xylose lysine deoxycholate modified agar *Salmonella Shigella* agar	37°C – 24 h 37°C – 24 h
*Listeria monocytogenes*	ISO 11290–1:1996	PALCAM Agar Listeria Listeria chromogenic agar base	37°C – 24 h 37°C – 24 h

The results were expressed as colony-forming units per gram of cheese. *Salmonella* and *Listeria* were reported as either not detected or detected in 25 g. According to Public Health Laboratory Service (PHLS) guidelines [35], the microbiological quality of ready-to-eat foods, including dairy products, can be classified as satisfactory, acceptable, unsatisfactory, and unacceptable/potentially hazardous based on their bacterial counts (Table-3).

**Table-3:** Microbiological limits of dairy products expressed in CFU as set by PHLS.

Bacterial strain	Satisfactory	Acceptable	Unsatisfactory	Unacceptable/potentially hazardous
Total *Coliforms* 30°C	<20	20–10^4^	>10^4^	NA
Escherichia coli *44°C*	<20	20–<100	≥100	NA
Staphylococcus aureus 37°C	<20	20–<100	100–<10^4^	≥10^4^
*Listeria monocytogenes* 37°C	Not detected in 25 g			Detected in 25 g
*Salmonella* 37°C	Not detected in 25 g			Detected in 25 g

CFU=Colony-forming unit , PHLS=Public Health Laboratory Service

### Antibiotic susceptibility test

The susceptibility of identified isolates to antimicrobial drugs commonly prescribed in the Lebanese community [36] and those used for treating infections caused by Gram-negative bacteria and listeriosis (amoxicillin-clavulanic acid [AMC] 20/10 mg, cefixime [CFM] 5 mg, levofloxacin [LEV] 5 mg, ciprofloxacin [CIP] 5 mg, azithromycin [AZM] 15 mg, doxycycline [DO] 30 mg, aztreonam [ATM] 30 mg, chloramphenicol [C] 30 mg, E 15 mg, S 300 mg, and SMT 250 mg) was detected in this study on several cheese samples by chromatographic analysis. The disk diffusion method outlined by the Clinical and Laboratory Standard Institute (CLSI, 2017) was used to test antibiotic susceptibility [37]. Briefly, isolates were diluted in normal saline solution 0.85% to a density of 0.5 McFarland turbidity standard. A 100 mL of subculture was streaked on the surface of Mueller-Hinton agar (Biomaxima, Poland). Antibiotic disks (Oxoid-UK, Bio-Rad-USA) or prepared antibiotic solutions were either placed on the surface of the agar or in the wells formed within the agar and incubated at 37°C for 24 h. Furthermore, according to CLSI (2017) guidelines [37], zone diameters were measured and classified into resistant, intermediate, or susceptible. *E. coli* ATCC 25922 strain was used as a control strain. Resistance to at least three classes of antibiotics identifies the isolate as multidrug-resistant bacteria [38].

### Antibiotic and pesticide residues

#### Chemicals and reagents

Antibiotics and pesticide residues were analyzed using analytic grade reagents. Standards of high purity grades (>99%) for 26 antibiotics belonging to four families: Sulfonamides (sulfacetamide, sulfisomidine, SMT, sulfapyridine, sulfamerazine, sulfameter, sulfachloropyridazine, sulfamethoxazole, sulfabenzamide, sulfadimethoxine, sulfaquinoxaline, sulfamethoxypyridazine, and sulfadoxine), quinolones (nalidixic acid, oxolinic acid, CIP, norfloxacin, ofloxacin, flumequine, danofloxacin, enrofloxacin, lomefloxacin, and sarafloxacin), tetracyclines (tetracycline and oxytetracycline), and β-lactams (ampicillin) in addition to a hundred pesticide standards were procured from Sigma-Aldrich. Each pesticide stock solution was accurately prepared in acetonitrile at a concentration of 1 g/L. The working standards were freshly prepared by diluting the stock solution in acetonitrile to the desired concentration. Both stock and working solutions were stored at −20°C.

High-performance liquid chromatography (HPLC)-grade water, HPLC-grade acetonitrile, disodium ethylenediaminetetraacetate dihydrate (Na_2_ EDTA), and magnesium sulfate (MgSO_4_) were also supplied by Sigma-Aldrich.

### Sample preparation

#### Sample extraction for antibiotic residues analysis

An optimized multiclass method was employed for the detection and quantification of antibiotics in cheeses using liquid chromatography coupled to mass spectroscopy.

In a centrifuge tube, 10 g of homogenized samples were weighed, and 1 mL of chelating agent EDTA was added to prevent the loss of tetracycline antibiotic by complexing to the cations found in the extraction solution [39]. After 1 min of vigorous shaking, 4 g of magnesium sulfate, 1 g of sodium chloride, and 0.5 g of sodium citrate dibasic sesquihydrate were added to the tubes. Later, the tubes were centrifuged at 2404× *g* at 4°C for 10 min and then placed at −20°C for 2 h. Subsequently, 50 mg of primary secondary amine, 100 mg C18, and 150 mg of MgSO_4_ were mixed with 1 mL of the aqueous supernatant and shaken vigorously for 3 min before being centrifuged at 2404× *g* at 4°C for 5 min. Finally, the solution was filtered through a 0.22 μm polyvinylidene fluoride (PVDF) filter for further liquid chromatography with tandem mass spectrometry (LC–MS/MS) analysis.

#### Sample extraction for pesticide residues analysis: QuEChERS extraction

A modified QuEChERS approach based on EN 15662 method was employed for the analysis of pesticide residues in the cheese samples [40]. Briefly, 10 mL of acetonitrile was added to a 10 g homogenized cheese sample and shaken vigorously before adding the extraction salts (4 g of MgSO_4_, 1 g of sodium chloride, and 0.5 g of sodium citrate dibasic sesquihydrate) to the extraction tube. Later, the tubes were centrifuged at 2404× g at 4°C for 10 min and then placed at −20°C for 2 h.

Furthermore, for the clean-up step, an aliquot of 1 mL of acetonitrile phase was transferred to dispersive enhanced matrix removal (EMR) and shaken vigorously for 3 min, and centrifuged at 2404× *g* at 4°C for 5 min. The extract was isolated and filtered through a 0.22 mm PVDF filter for further LC–MS/MS and GC–MS/MS analysis.

### Liquid chromatography with tandem mass spectrometry equipment

Antibiotic and pesticide residue analysis was performed using Shimadzu LC-MS-8045. Electron spray ionization was used for mass spectroscopy detection with data acquisition in multiple reaction monitoring (MRM) mode (ion spray voltage: 4 kV, nitrogen for desolvation, and dried gas: 10 L/min). The MRM parameters for each antibiotic tested are summarized in Table-4.

**Table-4:** Multiple reaction monitoring acquisition conditions for each antibiotic used.

Antibiotics	Precursor ion (m/z)	Product ion (m/z)	Collision energy (eV)	Retention time (min)
Sulfonamides				
Sulfacetamide	214.9	92	30	2.971
Sulfisomidine	279.2	124.1	20	3.973
Sulfamethazine	279.2	186.1	17	4.048
Sulfapyridine	250	156.1	27	4.657
Sulfamerazine	265.2	156.15	15	4.967
Sulfameter	280.95	156.05	17	5.649
Sulfachloropyridazine	285.1	156.15	15	5.904
Sulfamethoxazole	253.95	156.1/92.1	45	6.091
Sulfabenzamide	277	156	13	6.711
Sulfadimethoxine	311.2	156	20	7.392
Sulfaquinoxaline	301	156.05	17	7.705
Sulfamethoxypyridazine	280.95	156.05	16	5.717
Sulfadoxine	311.2	156	20	7.389
Tetracyclines				
Tetracycline	445.1	410.25	20	5.341
Oxytetracycline	461.1	443.3	14	5.47
Quinolones				
Ofloxacin	362.1	318.2	19	5.153
Norfloxacin	320.1	302.15	21	5.339
Ciprofloxacin	332.1	314.2	21	5.404
Enrofloxacin	360.1	316.3	19	5.527
Danofloxacin	358.1	340.2	24	5.591
Lomefloxacin	352.1	265.15	22	5.657
Oxolinic acid	262.2	244.1	17	7.772
Sarafloxacin	386.1	368.25	21	5.905
Nalidixic acid	233	215.1	13	8.764
Flumequine	262	244.1	16	8.833
β-lactams				
Ampicillin	350.3	106.05	16	14.423

Antibiotic residues were separated using a C18 analytical column (Shim-pack GIST, 2.1 mm inner diameter × 100 mm length, 3 μm particle size; Japan), and pesticide residues were separated using a C18 analytical column (DB-5MS, 0.25 mm inner diameter × 30 m length, 0.25 μm particle size; Agilent Technologies, Santa Clara, CA, USA).

Antibiotics and pesticide residues were separated at 40°C with an injection volume of 10 μL and flow rate of 0.3 and 0.2 mL/min, respectively. The solvents used as mobile phases for antibiotic residue separation were (A) 5 mM ammonium acetate in water and (B) methanol. The gradient elution program was as follows: 30% B for 2 min, 90% B for 10 min, 90% B for 12 min, and 10% B for 12 min and 10 s. The final run time of the method was 16 min. Similarly, the mobile phase for pesticide residues was composed of (A) 5 mM ammonium acetate in water and (B) methanol. The gradient program for elution started with 40% B for 6 min, then increased to 50% for 2 min and continued to increase to 55% for 9.5 min. At 30 min, the concentration of B becomes 95%, then decreased to 15% at 30.01 s until it stops at 40 min.

### Liquid chromatography with tandem mass spectrometry parameters

The GC–MS/MS analyses were performed using Shimadzu GC-MS-TQ8040 NX (Kyoto, Japan), equipped with an AOC-20s autosampler. The separation was carried out with the HP-5MS column (Agilent Technologies, Santa Clara, CA, USA), 0.25 mm × 30 m, 0.25 mm, and helium (purity 99.996%) was used as a carrier gas at a constant pressure of 151.9 kPa. The splitless injection mode was used, and the injection temperature was set to 250°C. The total run time was 24.33 min, during which the column temperature increased from 90°C (hold time 1 min) to 130°C (rate 30°C/min) and then 330°C (hold time 2 min and rate 10°C/min.). The mass spectrometer was set to operate with the following parameters (electron energy = 70 eV, solvent delay = 4.5 min, and the temperatures of the source, transfer line, and quadrupole temperatures were = 280°C and 150°C, respectively) with an electron impact source in MRM modes.

### Verification procedure

In-house method verification was performed according to the requirements of the European Union (EU) Commission Decision 2002/675/EC [41]. The recovery of antibiotic and pesticide residues was estimated in milk and was calculated as a ratio between the determined (analyzed) concentration and the real (spiked) concentration. Blank matrixes were spiked at MRL concentrations (100 ppb for all antibiotics tested except for ampicillin, 50 ppb), whereas pesticide recovery matrixes were spiked at 10 μg/kg and 50 μg/kg. The spiked and blank samples were then analyzed by LC–MS/MS as described previously.

The MRL values for milk established by the European Commission were adopted in this study because no MRL values were established for cheeses, and cheeses are mainly made of milk. Thus, any residue detected in cheese originates from milk.

### Statistical analysis

The collected data were analyzed using Microsoft Excel 2016 and the Statistical Package for the Social Sciences (IBM SPSS statistics 22); statistical significance was assumed when p < 0.05. Pearson’s Chi-square test was used for analysis.

## Results

### Microbial count of dairy products

The non-conformity of the dairy product sample was evaluated according to the Public Health Laboratory Services standards (PHLS) relative to the microbiological quality of ready-to-eat foods.

**Figure-1:**
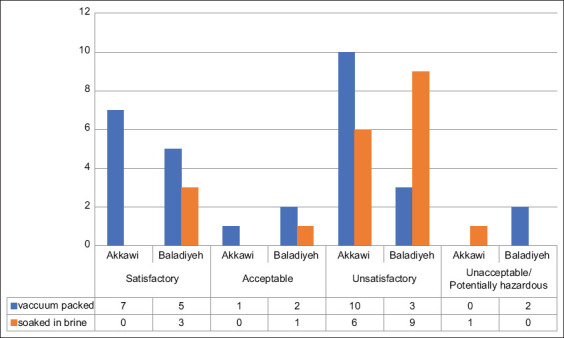
Microbiological classification of cheese samples according to cheese type and packaging.

In comparison to PHLS, 56% of the 50 samples tested were of unsatisfactory microbiological quality, 30% were satisfactory, 8% were acceptable, and 6% were unacceptable/potentially hazardous (Table-5). Furthermore, two out of three of the unacceptable/potentially hazardous samples tested positive for *Listeria*, while the third sample was contaminated with *S. aureus* more than the set limit. A significant correlation (p < 0.05) was found between the microbiological classification of Akkawi cheese samples and packaging status, that is, samples of Akkawi that were microbiologically satisfactory and acceptable were all individually vacuum packed, whereas unsatisfactory and unacceptable samples were individually vacuum packed or sold unpacked and soaked in brine (Figure-1). However, no significant correlation was observed in Baladiyeh cheese between microbiological classification and the packaging status of tested samples. Moreover, no statistical correlation was discovered between the microbiological classification of the samples tested and the presence or absence of antibiotic residues in the cheese sample.

**Table-5:** Microbiological classification of tested cheese samples according to PHLS.

Sample	Satisfactory (n/%)	Acceptable (n/%)	Unsatisfactory (n/%)	Unacceptable/potentially hazardous (n/%)
All samples	15 (30)	4 (8)	28 (56)	3 (6)
Baladiyeh	8 (32)	3 (12)	12 (48)	2 (8)
Akkawi	7 (28)	1 (4)	16 (64)	1 (4)

PHLS=Public Health Laboratory Service

### Antibiotic susceptibility

Table-6 displays the results of the susceptibility testing performed according to CLSI guidelines. All *E. coli* isolates tested positive for E and SMT resistance. DO resistance was found in a high percentage of tested *E. coli* (93%, 27/29). Frequent resistance was found against AMC (69%, 20/29) and AZM (59%, 17/29). Resistance was also observed to CFM (38%, 11/29), C (31%, 9/29), CIP (24%, 7/29), and LEV (24%, 7/29) which were observed. S (93%, 27/29), ATM (86%, 25/29), LEV (76%, 22/29), and C (69%, 20/29), on the other hand, showed a high percentage of susceptibility.

**Table-6:** Antibiotic susceptibility and resistance (n/%) of *E. coli* isolated from Akkawi and Baladiyeh cheese.

Antibiotic	*E. coli* isolates (n = 29)		

	R (n/%)	I (n/%)	S (n/%)
AMC 20/10 µg	20 (69)	5 (17)	4 (14)
ATM 30 µg	4 (14)	3 (10)	22 (76)
AZM 15 µg	17 (59)		12 (41)
CFM 5 µg	11 (38)	8 (28)	10 (34)
C 30 µg	9 (31)	1 (3)	19 (66)
CIP 5 µg	7 (24)	6 (21)	16 (55)
DO 30 µg	27 (93)	2 (7)	
E 15 µg	29 (100)		
LEV 5 µg	7 (24)		22 (76)
S 300 µg	2 (7)	2 (7)	25 (86)
SMT 250 µg	29 (100)		

*E. coli=Escherichia coli,* AMC=Amoxycillin-clavulanic acid, ATM=Aztreonam, AZM=Azithromycin, CFM=Cefixime, C=Chloramphenicol, CIP=Ciprofloxacin, DO=Doxycycline, E=Erythromycin, LEV=Levofloxacin, S=Streptomycin, SMT=Sulfamethazine

All tested *E. coli* isolates were labeled as multidrug resistant, indicating that resistance to three or more antimicrobial agents was observed. Furthermore, as shown in Table-7, six isolates were found resistant to three antibiotics, five isolates were found resistant to four antibiotics, while other isolates were found resistant to up to 10 antibiotics.

**Table-7:** Antibiotic resistance profile of *E. coli* isolates.

Antibiotics count	Antibiotic	Number of isolates
3	E, SMT, DO	5
3	E, SMT, AMC	1
4	E, SMT, DO, AMC	2
4	E, SMT, DO, ATM	1
4	E, SMT, DO, AZM	1
4	E, SMT, DO, C	1
5	E, SMT, DO, AMC, CFM	2
5	E, SMT, DO, AMC, AZM	3
5	E, SMT, DO, AZM, CFM	1
6	E, SMT, DO, AMC, AZM, CFM	1
6	E, SMT, AMC, AZM, CFM, S	1
6	E, SMT, DO, AMC, AZM, C	1
6	E, SMT, DO, AMC, AZM, C, CIP	1
7	E, SMT, DO, AMC, AZM, CFM, LEV	1
7	E, SMT, DO, AMC, AZM, CIP, LEV	1
8	E, SMT, DO, AMC, AZM, C, CIP, LEV	1
9	E, SMT, DO, AMC, AZM, CFM, C, CIP, S	1
9	E, SMT, DO, AMC, AZM, CFM, C, CIP, LEV	1
9	E, SMT, DO, AMC, AZM, CFM, C, LEV, ATM	1
10	E, SMT, DO, AMC, AZM, CFM, CIP, C, LEV, ATM	2

*E. coli=Escherichia coli*, AMC=Amoxycillin-clavulanic acid, ATM=Aztreonam, AZM=Azithromycin, CFM=Cefixime, C=Chloramphenicol, CIP=Ciprofloxacin, DO=Doxycycline, E=Erythromycin, LEV=Levofloxacin, S=Streptomycin, SMT=Sulfamethazine

### Chromatographic analysis

#### Method performance

Liquid chromatography with tandem mass spectrometry method performance for antibiotic residues

**Figure-2:**
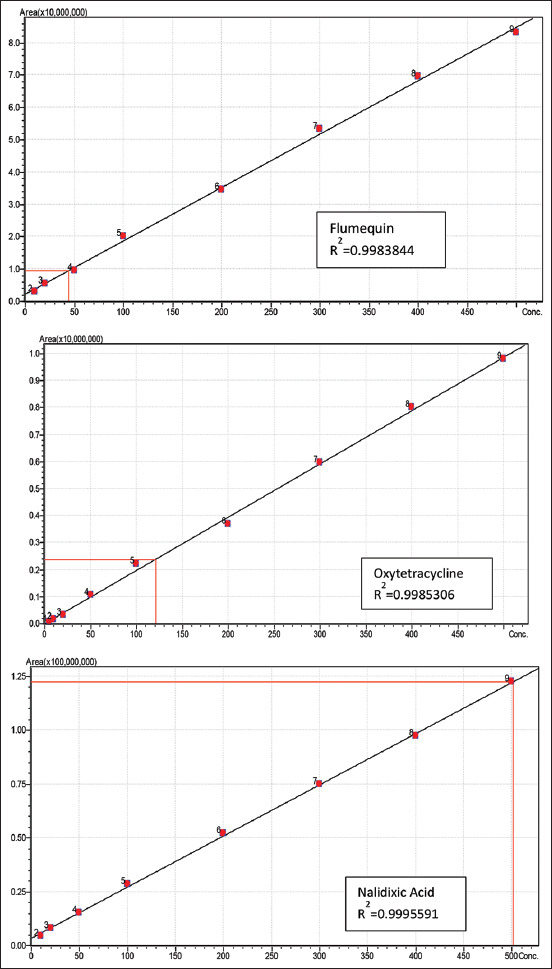
Calibration curve for flumequin, oxytetracycline, and nalidixic acid at 9 different concentration points

The method was validated in accordance with the EU commission decision 2002/675/EC criteria. Specificity, recovery, linear range, repeatability, reproducibility, accuracy, and limit of quantification (LOQ) were determined based on these requirements. Calibration curves were plotted with nine concentration levels ranging from 5 ppb to 500 ppb (Figure-2). Good linearity was obtained from calibration curves with a high correlation coefficient (R^2^ > 0.995).

Table-8 summarizes the precision: Recovery, relative standard deviation (% RSD), and LOQ. Except for ampicillin (50 μg/kg), the values obtained were calculated using spiking standards equal to the antibiotic MRL of 100 μg/kg. The calculated mean recoveries ranged between 81 and 119%, which is within the acceptable range recommended by AOAC, 2002 [42]. A signal-to-noise ratio of 10:1 was used to determine LOQ, which was found to be equal to 10 μg/L.

**Table-8:** MRL set by EU, mean recoveries, STDEV, RSD of repeatability, and LOQ for a number of antibiotics tested.

Antibiotics	MRL μg/kg in milk	Mean recoveries (%)	STDEV	RSD (%)	LOQ μg/L
Sulfonamides					
Sulfisomidine	100	89	6.29	7.06	10 μg/L
Sulfamethazine	100	81.42	3.88	4.77	
Sulfameter	100	92.16	4.47	4.85	
Sulfachloropyridazine	100	104.24	6.79	6.52	
Sulfamethoxypyridazine	100	89.45	3.15	3.52	
Sulfapyridine	100	94.76	14.64	15.46	
Sulfadimethoxine	100	93.056	3.76	4.04	
Sulfamethoxazole	100	110.35	6.71	6.1	
Sulfabenzamide	100	104.7	8.7	8.3	
Sulfaquinoxaline	100	85.75	5.03	5.87	
Sulfadoxine	100	93.06	3.76	4.04	
Tetracyclines					
Tetracycline	100	85.275	7.32	7.69	
Oxytetracycline	100	84.6	9.77	11.55	
Quinolones					
Enrofloxacin	100	119	14.8	12.43	
Danofloxacin	100	116.97	12.50	10.69	
Lomefloxacin	100	96.79	7.05	7.29	
Sarafloxacin	100	82.8	9.45	11.42	
β-lactams					
Ampicillin	50	127.73	2.55	4	

STDEV=Standard deviation, RSD=Relative standard deviation, LOQ=Limits of quantification, MRL=Maximum residue limit, EU=European Union

### Liquid chromatography with tandem mass spectrometry method performance for pesticide residues

Recovery experiments were conducted to evaluate the accuracy and precision of all pesticide residues. Two spiking values 10 μg/kg and 50 μg/kg of five replicates were used for this purpose. All calculated mean recoveries were between the acceptable range of 70 and 120%, and the calculated RSD of repeatability for each pesticide was <20%.

**Figure-3:**
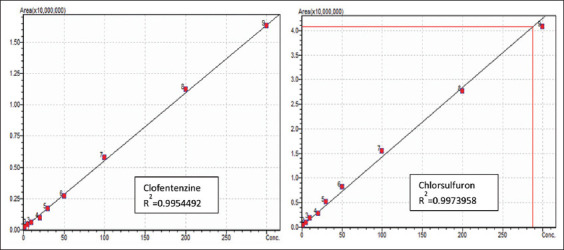
Calibration curves for chlorsulfuron and clofentezine at 9 different concentrations.

The calibration curves formed from nine concentration standards were used to assess the response linearity (Figure-3). The obtained curves were linear and had a high correlation coefficient (R^2^ > 0.995). The mean recoveries, % RSD, and R^2^ values were in accordance with the performance criteria specified in SANCO/12495/2011 [43].

Occurrence of antibiotic and pesticide residues in cheese samples

Eight samples (14%) of the 50 cheese samples tested were contaminated with antibiotic residues, but none of the samples contained more than 1 antibiotic. Eight samples were contaminated with SMT. All contaminated samples contained SMT at levels lower than the MRL (100 μg/kg) set by the European Commission, with results ranging between 13 and 31 μg/kg. Furthermore, five samples were found to be containing SMT between LOQ and LOD (LOQ/3). Thus, the percentage of positive samples (>LOQ) was 14%. Table-9 summarizes the presence of antibiotics in the cheese samples tested. However, none of the cheese samples were contaminated with any pesticide of the hundred pesticides tested.

**Table-9:** Occurrence of sulfonamides, tetracyclines, quinolones, and β-lactams in cheese samples tested.

Cheese samples, n = 50	Sulfonamides	Tetracyclines	Quinolones	β-lactams

	Sulfamethazine			
Mean (μg/L)	20.9* ± 0.71	0	0	0
Min (μg/L)	0	0	0	0
Max (μg/L)	31	0	0	0
*n* positive	8	0	0	0
% positive	14	0	0	0

* Mean value of eight contaminated samples

## Discussion

Traditional cheeses, such as Baladiyeh and Akkawi cheese, are exposed to different types of contamination during their manufacturing process. The use of unpasteurized milk containing pathogenic bacteria and manual handling at various stages of preparation are the main sources of contamination [28]. The use of unpasteurized milk in Baladiyeh cheese is still permitted by some Lebanese producers who lack good manufacturing practices knowledge, work informally, and are not registered with the relevant competent authorities. The two tested kinds of cheese had similar microbiological results, probably due to the similarities in their chemical characteristics, as both types of cheese have high moisture content, with the Lebanese Standards Institution (LIBNOR) requiring maximum moisture content of 56% for full fat salted Akkawi cheeses and 60% full fat salted Baladiyeh cheese [44, 45]. Such white Mediterranean cheeses are stored in brine with high salt content [46], and recent studies revealed that brine salt concentration affects the growth of common bacteria found in dairy products by disturbing the bacterial cell membrane, viability, and proteinase activity [47]. Despite the limited number of studies on the microbiology of cheeses matured in brine, it was discovered that the brine salt concentration is critical in controlling the type and number of bacterial flora found in cheese matured in brine [48]. In Lebanon, limited studies evaluating the microbiological quality of food categories revealed that dairy-based products are among the most contaminated food categories, with foodborne pathogenic bacteria such as *L. monocytogenes*, *S. aureus*, and *E. coli* present in food and dairy samples [26, 28, 49, 50].

The results in this study are consistent with the previous findings in which foodborne pathogenic bacteria (mentioned previously) were detected in the samples tested in this study. About 70% of the samples (35 out of 50) tested positive for *Coliforms*, and more than half of the samples (26 out of 50) were unsatisfactory, and 52% of samples were contaminated with *E. coli* above the allowed limits. Total *Coliforms* reflect the presence of fecal contamination and the absence of hygienic practices during and post-production stages [51]. Furthermore, water used during dairy product preparation can be a source of microbial contamination [52]. In contrast, testing for the presence of *L. monocytogenes* indicates the safety status of the sample and determines whether it is fit for human consumption. According to PHLS guidelines and LIBNOR, two samples tested positive for *L. monocytogenes* and were considered unacceptable for human consumption.

Antimicrobial resistance was ever linked to hospitals and health-care providers. Despite that, recent studies have revealed that food-producing animals form a powerful source of multidrug-resistant organisms [53, 54]. Worldwide, AMR is constituting a major challenge for health-care providers due to the appearance of multidrug resistance in Gram-negative bacteria [55] and the drying up of antibiotics pipeline where resistance to traditional drugs is increasing and newer drugs are also experiencing resistance [56]. *Escherichia coli* resistance to antimicrobial agents is of special interest since that *E. coli* is the most common Gram-negative bacteria in humans causing urinary tract infections, diarrhea, and a common cause of hospital- and community-acquired bacteria [57, 58]. In Lebanon, very few studies were conducted to determine AMR profiles for isolates other than clinical ones. Surprisingly, *E. coli* isolated from cheese samples tested in this study were all found to be resistant to at least three antibiotics: E 100%, SMT 100%, and DO 93% (Table-7). Recent studies regarding drug resistance of *E. coli* have shown that macrolides are not effective against the *Enterobacteriaceae* family and *E. coli* possess high resistance to E [59] in addition to that high rates of *E. coli* resistance (more than 90%) observed against SMT [60]. *Escherichia coli* resistance to AMC, S, CIP, E, tetracyclines, and C was also reported in several studies conducted on artisanal cheeses in Iran, Egypt, Spain, and Mexico [61–64]. The results of this study were consistent with the results of a study done on *E. coli* isolates from Lebanese dairy products where all isolates were found to be resistant to at least one antimicrobial agent [26]. Similarly, a study on fresh cheeses in Mexico revealed that all isolated diarrheagenic *E. coli* pathotypes were resistant to at least five antibiotics [64]. *Escherichia coli* isolates tested in this study showed high resistant prevalence against antibiotics that are commonly prescribed among the Lebanese community (AMC 69%, AZM 59%, DO 93%, CIP 24%, LEV 24%, and CFM 38%). These alarming results may be due to the uncontrolled and excessive use of antibiotics by the Lebanese community and indiscriminate treatment for animal husbandry.

Regarding antibiotic and pesticide residues, there is a lack of data about their presence in food of animal origin. Limited studies were conducted in Lebanon to assess the antibiotic residues in milk, beef, and poultry [65–67]. However, no information has been reported on evaluating veterinary drug residues in cheese in Lebanon. This study showed the absence of pesticide residues in all cheese samples tested and the presence of SMT residues in 14% of the tested samples. A recent study conducted in Lebanon to determine the level of contamination with antimicrobial residues in chicken, beef, and milk showed that among 99 milk samples tested, 34 samples were contaminated with antimicrobial agents. Furthermore, according to Mokh *et al*. [65], sulfonamides were the most detected family, and SMT was found to be one of the most detected residues in the matrixes tested. Besides from the negative effects on human health, the presence of antibiotic residues in milk can affect the cheese making processes, resulting in a flavor change [22]. The presence of SMT residues or other undesirable antibiotic residues in dairy products could be due the result of abusive veterinary drug treatment that did not adhere to the safety requirements for quantities and withdrawal times [68, 69].

## Conclusion

To the best of our knowledge, this is the first study conducted in Lebanon to assess both the chemical and microbiological status of dairy products commercialized in the local market. The chromatographic analysis of cheese samples revealed antibiotic residues in 14% of samples, which cause several health problems such as resistance development, allergic reactions, and toxicity. Furthermore, due to poor microbiological quality and the presence of multidrug-resistant strains, which pose a real threat to public health and are a potential source of foodborne diseases, extreme caution is required. This suggests that antimicrobial agents are being misused in Lebanon, where veterinary prescriptions are not mandatory and withdrawal times are not followed. As these problems worsen, the rational use of antimicrobial agents must be emphasized through official legislation, public awareness programs, and regular antimicrobial susceptibility surveillance. Furthermore, the obtained data will be used to advocate for the prompt implementation of the enacted Lebanese food safety law and the activation of the Lebanese Food Safety Commission to establish a national surveillance system for AMR and tracking and identifying foodborne pathogens causing foodborne outbreaks and establishing inspection and programs and control measures to minimize risks for consumers.

## Authors’ Contributions

HE: Collected samples, bought material and reagents, performed the experiments, analyzed and interpreted the data, and wrote the manuscript. MK: Supervised, conceived, and designed the experiments, and review – edited the drafts of the manuscript. RH: Performed microbiological experiments. RJ: Did chromatographic experiments. AJ: Contributed reagents, materials, analysis tools, performed chromatographic analysis experiments, and review – edited the drafts of the manuscript. NE: Supervised, conceived, and designed the experiments, and review – edited the drafts of the manuscript. All authors have read and approved the final manuscript.
